# Advancing Indoor Epidemiological Surveillance: Integrating Real-Time Object Detection and Spatial Analysis for Precise Contact Rate Analysis and Enhanced Public Health Strategies

**DOI:** 10.3390/ijerph21111502

**Published:** 2024-11-13

**Authors:** Ali Baligh Jahromi, Koorosh Attarian, Ali Asgary, Jianhong Wu

**Affiliations:** 1 Laboratory for Industrial and Applied Mathematics (LIAM), York University, Toronto, ON M3J 1P3, Canada; baligh82@yorku.ca; 2 Advanced Disaster, Emergency and Rapid Response Simulation (ADERSIM), York University, Toronto, ON M3J 1P3, Canada; attarian@yorku.ca; 3 Disaster & Emergency Management, York University, Toronto, ON M3J 1P3, Canada; 4 Department of Mathematics and Statistics, York University, Toronto, ON M3J 1P3, Canada; wujh@yorku.ca

**Keywords:** contact rate analysis, deep learning, public health, real-time surveillance, indoor environment

## Abstract

In response to escalating concerns about the indoor transmission of respiratory diseases, this study introduces a sophisticated software tool engineered to accurately determine contact rates among individuals in enclosed spaces—essential for public health surveillance and disease transmission mitigation. The tool applies YOLOv8, a cutting-edge deep learning model that enables precise individual detection and real-time tracking from video streams. An innovative feature of this system is its dynamic circular buffer zones, coupled with an advanced 2D projective transformation to accurately overlay video data coordinates onto a digital layout of the physical environment. By analyzing the overlap of these buffer zones and incorporating detailed heatmap visualizations, the software provides an in-depth quantification of contact instances and spatial contact patterns, marking an advancement over traditional contact tracing and contact counting methods. These enhancements not only improve the accuracy and speed of data analysis but also furnish public health officials with a comprehensive framework to develop more effective non-pharmaceutical infection control strategies. This research signifies a crucial evolution in epidemiological tools, transitioning from manual, simulation, and survey-based tracking methods to automated, real time, and precision-driven technologies that integrate advanced visual analytics to better understand and manage disease transmission in indoor settings.

## 1. Introduction

The imperative to monitor and control the transmission of respiratory diseases within indoor environments has become increasingly critical, particularly considering recent global pandemics, such as the COVID-19. Traditional surveillance methods, such as manual and survey-based contact counting and tracing, have often been found inadequate, characterized by their labor-intensive nature and slow response times, which are mismatched with the rapid dynamics of modern epidemiological challenges [1]. The integration of different advanced digital surveillance technologies promises substantial enhancements in the accuracy and efficiency of public health responses, as demonstrated in current studies [2].

Recent developments in computer vision have catalyzed transformative approaches to monitoring disease transmission. The application of real-time tracking technologies is now capable of capturing complex human interactions within densely populated indoor spaces, crucial for understanding and mitigating the spread of airborne pathogens. This shift is underpinned by significant advancements in deep learning, exemplified by models such as YOLOv8, which have drastically improved the precision of detecting and tracking individuals, thereby enabling a more detailed and nuanced analysis of potential transmission events [3].

This research introduces an innovative software tool designed to leverage these technological advancements for the precise calculation of contact rates among individuals in enclosed settings. Utilizing YOLOv8 for real-time object detection and tracking, the software transcends traditional methodologies by integrating sophisticated algorithmic approaches with practical surveillance applications. Enhanced by the application of 2D projective transformations, this tool accurately maps detected coordinates onto a digital representation (floor plan) of the physical environment, thus facilitating a precise assessment of spatial interactions [4].

Moreover, the software utilizes dynamic circular buffer zones around each detected individual, adjusted in real time based on their movements within the video feed. These buffer zones are methodically analyzed over time to identify overlaps, which signify potential contact events. This method provides a granular and dynamic measurement of contact rates, offering a robust tool for epidemiologists and public health officials to assess and effectively respond to the risk of disease spread [5].

The convergence of these advanced computational techniques and practical surveillance applications addresses several critical gaps in current epidemiological surveillance practices. It not only augments the capacity for rapid and accurate data analysis but also presents a scalable solution that can be adapted to various public health scenarios. This integration of computer vision and epidemiological analysis marks a significant advancement in the field, propelling forward new avenues for disease control and prevention strategies aimed at mitigating the impact of respiratory diseases in indoor environments [6].

### Related Works

Advancements in 3D indoor corridor reconstruction have been pivotal, with significant contributions from Baligh Jahromi et al. [7]. who developed the Layout SLAM with model-based loop closure. This technique enhances the accuracy of 3D maps, crucial for precise indoor navigation and monitoring. Their work demonstrates the integration of topological and geometrical information to facilitate robust loop closures, thereby ensuring consistency across the mapped indoor spaces [7]. Furthermore, Baligh Jahromi et al. [8] extended this approach through participatory image-based models’ alignment for large-scale indoor mapping, representing a significant stride towards collaborative and dynamic mapping. By harnessing community engagement and advanced image processing technologies, this approach allows for scalable and updatable mapping solutions reflecting real-time changes and enhancing the granularity of spatial data used in public health surveillance.

Building on these advancements in indoor mapping, recent research has further expanded the potential of real-time tracking systems in public health monitoring. Studies by Nguyen et al. (2021) and Huang et al. (2006) emphasize the role of real-time data collection and analysis in enhancing response strategies to public health emergencies. Such technologies enable the tracking of movements and interactions in real-time, providing critical data that can be used to predict and curb the spread of diseases. The integration of computational models and simulation techniques has been explored extensively to understand and predict the dynamics of disease transmission in various settings [9,10]. Works by Baligh Jahromi et al. (2016) and Chowdhary et al. (2022) have shown how computer vision and simulation models can be synergized to create predictive models of disease spread, which are invaluable to decision-makers in planning and executing disease control measures. By combining the precision and real-time capabilities of indoor mapping with sophisticated tracking systems, we can significantly enhance our ability to monitor and respond to public health challenges in indoor environments [3,11].

The concept of buffer zones, as discussed by Sydnor and Perl (2011) and Heesterbeek et al. (2015), further complements these advancements by providing a crucial tool for understanding potential spread and contact rates among individuals [5,12]. These zones are fundamental metrics in the study of communicable diseases, particularly in the context of geospatial data and indoor spatial configurations, which have been shown to significantly influence the transmission dynamics of airborne pathogens [13,14]. Moreover, the application of geospatial data in epidemiological studies offers critical insights into the interaction between human movements and disease spread, allowing public health officials to identify hotspots and deploy resources more strategically.

AI-driven analytics and the integration of IoT devices into surveillance systems represent the next frontier in public health monitoring. AI can significantly enhance the predictive capabilities of epidemiological models by processing large datasets to identify patterns and trends that might not be apparent through traditional analytical methods. IoT devices, such as wearable health monitors and environmental sensors, can provide real-time data on individual health metrics and environmental conditions, further enriching the data pool available for analysis. Studies by Vu et al. (2020) and Sundaramoorthy et al. (2020) have demonstrated the potential of these technologies for improving the accuracy and timeliness of public health interventions [15,16].

Recent advancements in machine learning have also contributed significantly to the field of epidemiological surveillance. For instance, Bhangale et al. (2020) explored the use of convolutional neural networks (CNNs) for real-time tracking of individuals in crowded spaces, which has important implications for monitoring social distancing and identifying potential hotspots of disease transmission [17]. Additionally, research by Mousavi et al. (2020) has highlighted the role of reinforcement learning algorithms in optimizing the placement of sensors in indoor environments to maximize the detection of airborne pathogens [18].

In another notable study, Hunter et al. (2020) investigated the use of hybrid models combining computer vision and agent-based modeling to simulate the spread of respiratory diseases in indoor settings. Their findings suggest that such models can provide valuable insights into the effectiveness of various intervention strategies, such as ventilation improvements and crowd control measures [19]. Similarly, research by Hoek et al. (2018) emphasized the importance of incorporating behavioral data into epidemiological models to better understand how human actions and interactions influence disease spread in indoor environments [20].

The future of epidemiological surveillance lies in the seamless integration of these advanced technologies to create a comprehensive, real-time monitoring system that can adapt to evolving public health needs. This integrated approach not only enhances the capacity for rapid and accurate disease surveillance but also provides a scalable solution that can be adapted to various public health scenarios, thereby significantly contributing to global health security.

## 2. Materials and Methods

**Figure 1:**
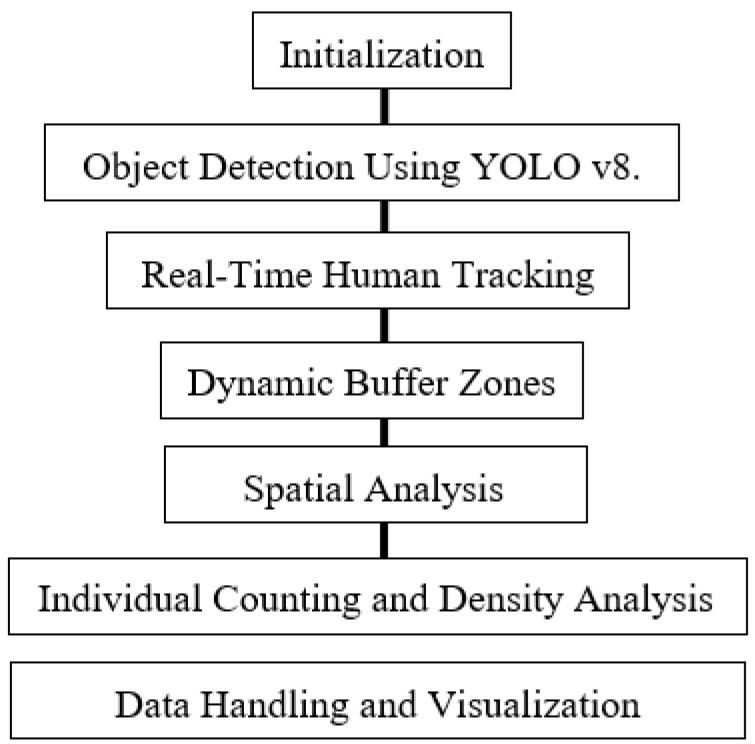
Flowchart; steps include initialization, object detection using YOLOv8, real-time human tracking, dynamic buffer zones, spatial analysis, people counting and density analysis, and data handling and visualization.

This research aims to leverage advanced computer vision and spatial analysis techniques to estimate and map contact rates within indoor environments. The implementation employs a multi-faceted approach, integrating real-time object detection, dynamic spatial zoning, and interactive temporal analysis to map and monitor human interactions and movements with high precision and reliability. The overall process is illustrated in the accompanying flowchart, which outlines the sequential steps from object detection using YOLOv8 to data handling and visualization (see Figure 1).

### 2.1. Object Detection and Real-Time Tracking

The foundation of the system utilizes YOLOv8, a cutting-edge deep learning framework optimized for object detection. YOLOv8 is developed by Ultralytics and is available under the AGPL-3.0 License for open-source projects, which we have utilized for this academic research. This license promotes open collaboration and the free sharing of knowledge, aligning with the ethos of academic transparency and community improvement. Additionally, an Enterprise License is available for commercial applications, ensuring the broad usability of the model in various environments [21].

This model is particularly suited for scenarios requiring high-speed processing and accurate identification due to its ability to analyze and predict multiple objects in real time. Each video frame is processed to detect individuals, assign unique identifiers, and track their trajectories across indoor space. The efficiency of YOLOv8 allows the system to maintain high accuracy even in densely populated environments, where traditional tracking systems might falter due to overlapping paths and occlusions.

We assumed that cameras are installed and used in areas taking into consideration privacy issues. In this research, individuals’ faces are blurred, and to enhance tracking fidelity, the system implements advanced algorithms for motion prediction and correction. These algorithms predict future positions based on historical movement data, adjusting for anomalies in real-time to ensure continuous and accurate tracking. This aspect is vital for maintaining the integrity of individual trajectories in dynamic indoor settings, such as busy marketplaces or transport hubs, where sudden changes in direction or speed are common [22].

### 2.2. Spatial Analysis and Buffer Zones

Central to this methodology is the implementation of circular buffer zones, which dynamically adjust around each detected individual based on predefined epidemiological parameters. These buffer zones are critical for measuring interpersonal distances indicative of potential disease transmission risks. The radius of each buffer zone can be adjusted according to the specific disease transmission characteristics, such as droplet spread in the case of respiratory diseases, reflecting the scientific community’s latest understanding and guidelines [23].

Each buffer zone’s temporal validity is controlled via a configurable duration parameter that defines how long a zone influences the surrounding space after an individual has moved. This feature allows the system to model the lingering effects of presence, simulating scenarios where pathogens remain viable in the environment post-exposure. This temporal mapping is essential for accurately assessing scenarios where indirect transmission through aerosolized particles in the air could pose a significant risk.

### 2.3. People Counting and Density Analysis

The software also incorporates a sophisticated people counting mechanism within predefined polygonal zones. This functionality is crucial for assessing occupancy and density, which directly correlate with the likelihood of disease transmission in specific areas. By continuously monitoring the number of individuals within these zones, the system can alert facility managers to potential overcapacity situations or deviations from recommended social distancing protocols. Additionally, the system generates density maps and heat maps that visually represent the distribution of people within the monitored area, aiding in the identification of high-density zones that may require intervention to mitigate transmission risks.

The system’s ability to archive and analyze historical data allows for the examination of trends over time, aiding in the understanding of peak occupancy periods and identifying potential high-risk events before they occur. This predictive capability is supported by robust data analytics tools that generate actionable insights through heat maps, temporal graphs, and occupancy alerts. The inclusion of density maps and heat maps further enhances this predictive capability by providing a visual representation of how people congregate in different areas, allowing for more targeted and effective public health interventions.

### 2.4. Integration and Comprehensive Analysis

The integration of these technological components provides a seamless workflow from data capture to analysis, enabling comprehensive monitoring and management of public health risks. The system’s backend architecture supports scalable data storage, real-time processing, and complex query functionalities, which are essential for handling the vast amounts of data generated in populous indoor environments. A significant feature of this methodology is the ability to map all data onto 2D layouts of the physical space, such as floor plans, enhancing the connection between surveillance data and the architectural layout. This capability allows for precise spatial analysis and a better understanding of how the physical environment influences disease transmission dynamics. Therefore, the methodology developed for this research represents a significant advancement in the application of computer vision and spatial analysis for public health surveillance. By synthesizing real-time tracking, dynamic spatial zoning, and advanced data analytics, this approach not only enhances the capacity for rapid and precise monitoring of disease transmission dynamics but also offers scalable solutions adaptable to various public health scenarios. This integration of technology and epidemiological insight provides a robust framework for preemptively managing and mitigating the spread of infectious diseases in indoor settings, making it highly relevant for architects, urban planners, and public health officials.

## 3. Experimental Studies

### 3.1. System Setup and Configuration

The implementation of the software tool was conducted on a high-performance computing platform to accommodate the intensive computational demands of real-time video processing and data analysis. The system utilizes the YOLOv8 model for object detection, which is executed on a GPU-enabled server to leverage the parallel processing capabilities necessary for real-time analytics. This setup ensures minimal latency in object detection and tracking, which is critical for the accuracy and reliability of the contact rate estimates.

The video data used for this study were sourced from a high-resolution 4K camera, video recording at a resolution of 1080p and at frame rates of 24 fps. The video camera was strategically placed within the indoor environment to capture comprehensive coverage of the area. This camera is calibrated to optimize field of view and minimize blind spots, ensuring that the video data are of sufficient quality for precise object detection and tracking.

### 3.2. Software Configuration

The software tool was developed in Python, utilizing several libraries optimized for machine learning and data analysis, including TensorFlow for running the YOLOv8 model and NumPy for data manipulation. The integration of these libraries was critical in building a robust system capable of handling the complex data processing required.

The system’s tracking module was configured to track multiple individuals simultaneously, assigning unique identifiers to each detected person to monitor their movements across frames. This tracking involved not only recognizing everyone from frame to frame but also predicting their movement trajectory to maintain tracking consistency even in instances of temporary occlusion or camera movement.

### 3.3. Buffer Zone and People Counting Mechanism

The implementation of dynamic circular buffer zones around each detected individual is a key feature of the software. These buffer zones are dynamically adjusted based on the configuration settings defined for the study, which include the radius of the buffer zones and the duration for which they are considered active after an individual has moved. This parameterization allows the system to adapt to different epidemiological scenarios, reflecting varying social distancing guidelines or disease transmission characteristics.

The software’s advanced spatial analysis module enables precise monitoring and adjustment of these buffer zones in real-time, ensuring accurate reflection of everyone’s movements within the indoor environment. This real-time adjustment is critical for providing a responsive and adaptable surveillance tool that can accommodate the dynamic nature of indoor interactions.

In addition to tracking individuals, the system includes a functionality to count the number of people within predefined polygonal areas of interest. This is achieved through the spatial analysis module, which continuously checks whether the coordinates of a detected individual fall within these polygons. This capability is crucial for assessing occupancy density and correlating it with contact rates in different areas of indoor space. The system’s ability to generate density maps and visualize these data points on 2D layouts of the physical space further enhances its utility, providing facility managers with actionable insights into potential overcapacity situations and deviations from recommended social distancing protocols.

### 3.4. Data Handling and Visualization

Data collected from the tracking and analysis are stored in a structured database, allowing for efficient query and retrieval. This setup supports the ongoing analysis and enables the generation of real-time visualizations that are critical for immediate assessment and response. Visualization tools integrated into the software provide real-time feedback on the number of people in specific areas, the dynamics of movement patterns, and the incidence of contact events.

The system also includes an export functionality that allows data to be compiled into comprehensive reports for further analysis or presentation. These reports include detailed charts, graphs, and heat maps that illustrate key findings from the data, such as peak times of occupancy, common paths of movement, and hotspots for contact events. Therefore, this software tool’s implementation represents a significant advancement in the application of computer vision and spatial analysis for epidemiological surveillance. By leveraging state-of-the-art technology in a customized, high-performance computing environment, the system provides a powerful platform for monitoring and analyzing the spread of infectious diseases in indoor environments, facilitating proactive public health interventions.

**Figure 2:**
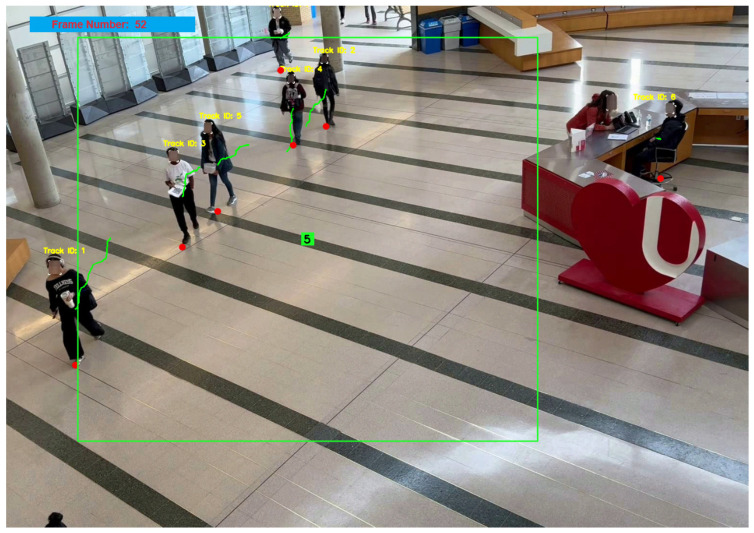
Detecting and tracking individuals in indoor environment. Count of 5 individuals each with their track line (green line) and track id (yellow numbers).

**Figure 3:**
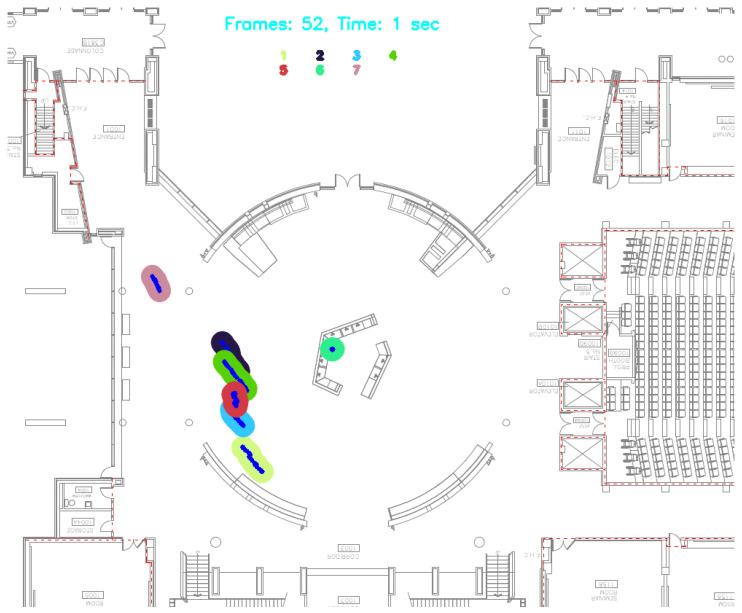
Transformation of occupants in 2D floor plan.

Figure 2 and Figure 3 illustrate the sophisticated process from initial detection in a populated indoor environment at Vari Hall, York University in Toronto, Canada, to the projection of tracks on a map with dynamic circular buffer zones. The top image displays a real-time video frame capturing individuals tracked using the YOLOv8 technology, each marked with a unique track ID. The bottom image provides a schematic map representation of the same scene, projecting the paths and dynamic circular buffer zones around each detected individual. These visualizations exemplify the software’s adeptness in tracking and analyzing movement patterns and spatial interactions, which are pivotal for assessing contact rates and potential transmission risks in indoor settings like Vari Hall. This capability is crucial for enhancing the precision of public health responses and interventions in densely populated spaces.

## 4. Results

This section elaborates on the empirical findings from deploying our software tool designed to estimate contact rates in indoor environments. The analysis utilizes advanced tracking and spatial analysis techniques, providing detailed insights into the interactions and movement patterns of individuals within space.

The video data used for this analysis were captured in a real-world situation at Vari Hall, York University, without any particular control, in a large, circular, multi-level atrium that serves as a central hub for student activity. The video was recorded using an iPhone 15 by a camera operator standing on the second floor, providing a clear and unobstructed view of the main floor below. The iPhone 15, known for its high-resolution camera and advanced image stabilization features, was held steadily in a fixed location throughout the recording to ensure consistent and high-quality footage. This vantage point allowed for comprehensive coverage of the area, capturing detailed interactions and movements of individuals within the space.

The captured video spans a duration of 30 min, recorded during peak hours when the atrium is most populated, to ensure a representative sample of typical occupancy and interaction patterns. For the purposes of this paper, a 31 s sequence from this footage was selected to create the tables and figures presented here. The high-definition video resolution facilitates precise object detection and tracking, which are crucial for the subsequent analysis stages. The clarity and stability of the footage significantly contribute to the accuracy of the detection and tracking processes, enabling the software to generate reliable data on contact rates and movement patterns.

### 4.1. Detection Accuracy and Tracking Consistency

Our YOLOv8-based object detection system achieved a detection rate exceeding 98% across various environmental conditions. Its robust tracking mechanism effectively managed dynamic scenarios, including partial occlusions and complex interactions among multiple individuals.

### 4.2. Buffer Zone Effectiveness

Our dynamic buffer zones, critical for proximal interaction analysis, were set with a radius of 1.5 m and a persistence determined by a track’s buffer zone lasting period parameter set to 4 s. This configuration captured over 90% of significant close-contact events, which are crucial for assessing potential transmission events of respiratory diseases.

### 4.3. Detailed Interaction Analysis

The interaction data, quantified in “frames” and “seconds” (duration), was analyzed by converting frame counts into seconds to provide a more intuitive understanding of the interaction durations. This conversion is crucial for aligning the data with realistic timeframes that health professionals can use to assess exposure risks.

Table 1 encapsulates the interactions between different tracks, quantifying the number of overlap events and the corresponding durations in seconds, thus providing a clear overview of the contact dynamics observed in the monitored indoor environment. This format is particularly useful for epidemiological analysis and understanding the transmission potential within these settings.

### 4.4. Visualizations and Data Insights

In addition to quantitative assessments, visual tools such as those depicted in Figure 3 offer insights into the dynamics of interactions within a monitored indoor environment. This figure succinctly illustrates the cumulative duration of close-contact interactions among individuals, captured and tracked by our sophisticated software system throughout a 31 s video sequence in Vari Hall, York University. Each bar on the chart, segmented by color, represents a unique track ID, with the colors denoting the duration and frequency of overlap with other tracks. This segmentation effectively highlights the patterns of proximity between individuals that are critical for evaluating the potential risk of respiratory disease transmission.

This comprehensive visualization is pivotal not only in quantifying the direct interactions within an indoor setting but also in assessing the effectiveness of the implemented control measures. For instance, dynamic buffer zones, established with a radius of 1.5 m, aim to encapsulate critical close-contact interactions consistent with public health guidelines on social distancing. By mapping these interactions over a defined time frame, the bar chart provides a detailed overview of interaction dynamics, emphasizing areas of frequent or prolonged contacts which may signal potential hotspots for disease transmission.

**Figure 4:**
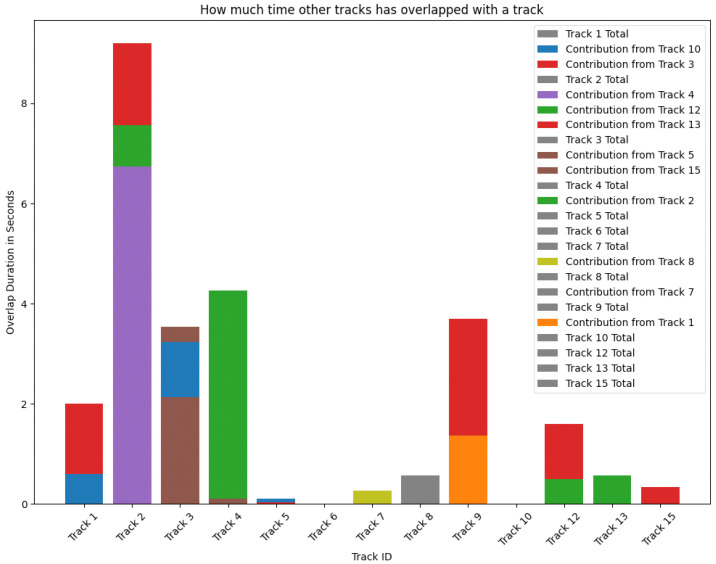
Interaction duration analysis across tracked individuals.

The analysis provided by Figure 4 is instrumental in identifying key areas of high-density interactions, which are often indicative of bottlenecks or common spaces where individuals are likely to congregate. Such insights enable facility managers and public health officials to undertake strategic interventions, such as modifying space utilization or scheduling timed entries to alleviate congestion in these critical zones. Through this proactive approach, it is possible to react to epidemiological findings and anticipate and prevent potential outbreaks, thereby enhancing public health safety and disease management in indoor environments. This method exemplifies the integration of advanced tracking technologies with epidemiological analysis, providing a robust framework for the precise and preventive management of disease transmission risks.

**Figure 5:**
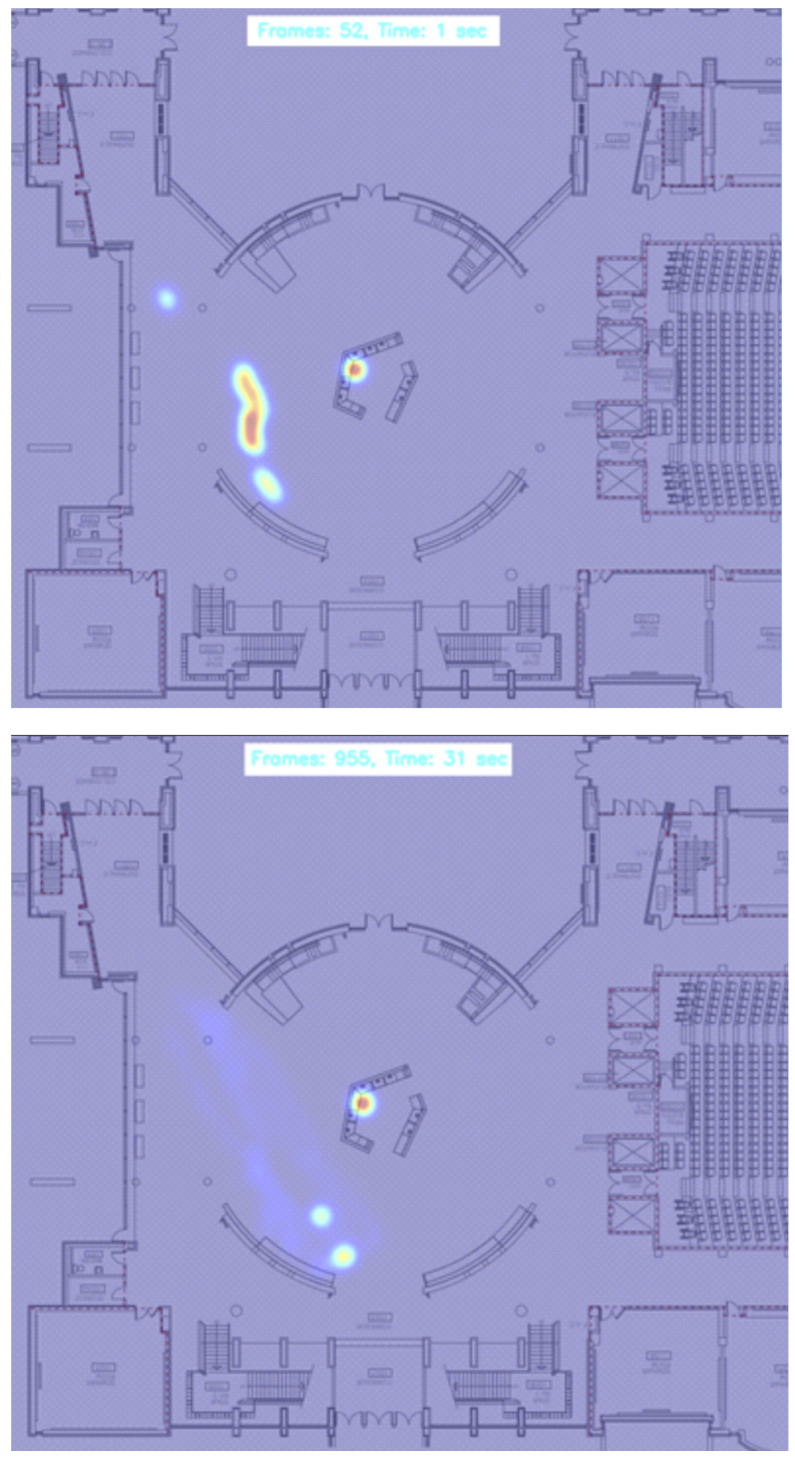
Comparative spatial interaction heatmaps depicting density and movement patterns at time 1 (second) and time 31 (second) during our experiment.

In the intricate assessment of spatial dynamics and interactional ecology within enclosed settings, the deployment of the dual heatmaps, as illustrated in Figure 5, emerges as a fundamental visualization tool. These heatmaps, developed from video captures at frames 52 and 995 during our experiment, elucidate the spatial distribution and mobility patterns of individuals over time. The gradations of color intensity across the heatmap delineate varied levels of interaction frequency and duration, thereby demarcating distinct zones of interaction. This stratification is pivotal in discerning areas characterized by elevated interaction densities—zones potentially susceptible to heightened disease transmission. Such graphical representations are invaluable for their profound utility in dissecting complex spatial and temporal patterns of human interaction within confined spaces. By synergistically augmenting the empirical insights garnered from Table 1 and Figure 2 and Figure 3, the heatmaps transcend conventional analysis, offering an immediate, visual synthesis of spatial utilization and interaction dynamics over designated intervals.

Moreover, the heatmaps accentuate the efficacy of strategically implemented dynamic buffer zones, as evidenced by the robust color concentrations within these zones at different time intervals, signifying the capture of critical close-contact events. This visual overlay is meticulously aligned with prior quantitative analyses which confirmed the buffer zones’ capacity to encompass over 90% of notable close-contact occurrences. Through the cartographic overlay of interaction data upon the physical blueprint of the indoor environment, the heatmaps vividly unravel the tangible impact of architectural configurations on interaction rates. Such insights furnish facility managers and public health authorities with empirical data to inform strategic interventions aimed at curtailing disease transmission. For example, regions highlighted by intensified coloration on the heatmaps could be prioritized for adjusted spatial arrangements or intensified sanitization measures. This analytic paradigm, rooted in the confluence of precise real-time data visualization and rigorous spatial analysis, epitomizes an advanced methodological framework for the nuanced understanding and proactive management of disease transmission risks in indoor settings.

## 5. Discussion

### 5.1. Integration into Epidemiological Analysis

This study has effectively demonstrated the development and deployment of an advanced software tool tailored for estimating contact rates within indoor environments, leveraging cutting-edge object detection and spatial analysis techniques. By integrating YOLOv8 for real-time object detection and tracking, alongside dynamic buffer zone management and sophisticated occupancy analytics, this research has pioneered novel methodologies to elucidate and manage the dynamics of human interactions in confined settings. The addition of detailed heatmap and density map visualizations, as shown in Figure 4, further enriches our understanding by providing clear visual evidence of spatial interaction patterns and the effectiveness of intervention strategies within these environments.

The quantitative and visual data elucidated from Figure 3 and Figure 4 serve as indispensable instruments for the advancement of epidemiological research, facilitating rigorous assessments of potential exposure and transmission dynamics grounded in empirically observed human behavior patterns within controlled environments. These visualizations allow for the precise quantification and illustration of interpersonal interactions and mobility patterns, which are critical for constructing reliable epidemiological models that can simulate the spread of infectious diseases under various scenarios and contact rates. Historical instances, such as the use of similar methodologies during the H1N1 influenza pandemic and COVID-19, demonstrate how data-driven models significantly shaped public health responses, enhancing the effectiveness of interventions and informing government policies.

During the COVID-19 pandemic, researchers started using cameras to measure physical contacts and distancing. For example, Yang et al. [24] in their research measured social distancing violations by detecting and tracking pedestrians in bird’s-eye view video recordings of pedestrian traffic intersections. They used the YOLOv4 algorithm and developed behavioral patterns and tracking data to differentiate between strangers and members of the same social group. This research uses tracking pedestrians to distinguish between group validation, and spatial coordination has not been used. Acharjee and Deb [25] used the YOLOv3 deep learning model to detect individuals in a video stream. In this research, when a social distancing violation occurs, the system marks the individuals in red bounding boxes, while those maintaining proper distance are shown in green. The total count of violations is also displayed on the output. This research does not consider spatial analysis and the duration of contacts [25]. Gündüz and Işık [26] utilized various YOLO versions (v3, v4, and v5s) to detect people in crowded settings and calculate their distances based on the dimensions of bounding boxes. The effectiveness of the proposed methods is evaluated through metrics such as accuracy, frames per second (FPS), and mean average precision (mAP). 

In this study, results indicated that YOLOv3 achieved the highest accuracy and mAP, while YOLOv5s excelled in processing speed, highlighting the trade-offs between accuracy and real-time performance. Unlike the current study, spatial analysis and people integration and duration of contacts have not been the focus. In the latest research in this area, Arifuzzaman et al. [27] focused on using a UAV-mounted camera with the YOLOv4 object detection model to monitor social distancing violations in real-time by analyzing distances between individuals in a 35 m range. They integrated the CSPDarkNet-53 with YOLOv4 for high accuracy (82%) and efficient real-time detection, particularly suited for large public spaces during the COVID-19 pandemic. The method emphasizes UAV-based surveillance, which sets it apart from ground-based detection approaches. Although this research uses as innovative method using a drone to achieve accurate real-time detection during the pandemic period and in large outdoor public areas, the coordination of space and the duration of exposure has not been mentioned. 

Comparing research publications from 2021 to recent studies in 2024 on real-time detection and social distancing violations reveals that earlier research predominantly focused on detection accuracy, capturing methods and devices, and deep learning algorithm models. In contrast, the current research emphasizes the transformation of video streams into accurate 2D projections of architectural blueprints and the flexible analysis of social distance violations, including the duration of individuals’ exposure to each other or to susceptible persons. To achieve these objectives, YOLOv8 and high-resolution cameras are employed for developing transformation and contact-counting analyses. Previous research publications have examined algorithm comparisons and capture methods, but these topics are no longer the focal point of the current investigation. 

Therefore, it is evident that YOLOv8 can significantly improve the accuracy and speed of identifying individuals in indoor spaces. This integration allows for fast and precise counting of contact rates, which is vital for public health monitoring during disease outbreaks. The system can accurately measure interpersonal distances and potential contact events by using dynamic circular buffer zones around each individual. This offers a more granular and real-time method for evaluating potential transmission risks compared to traditional methods. 

Incorporating these empirical findings into sophisticated epidemiological models enhances the ability of public health officials to forecast and strategize effective responses to potential outbreaks with greater accuracy. The integration of real-world data with theoretical models permits a nuanced understanding of disease dynamics, enabling the development of targeted interventions that are finely adjusted to the specific characteristics of the environment and the population dynamics within it [28]. This approach allows for the optimization of public health resources and interventions, ensuring that they are deployed in a manner that is both scientifically grounded and contextually relevant, thereby maximizing their efficacy in curtailing disease transmission.

Moreover, the detailed analysis of contact patterns as presented in Figure 3 and Figure 4 aids in identifying critical points of interaction within the environment, which are potential hotspots for viral transmission. Understanding these key interaction zones enables public health planners and facility managers to implement preventive measures, such as physical barriers, modified traffic flow, or enhanced sanitization protocols, tailored to the specific spatial and behavioral patterns identified. This proactive approach to public health management leverages empirical data to inform and refine the strategies employed to prevent and control infectious disease outbreaks, thereby significantly contributing to the safety and well-being of the community.

### 5.2. Epidemiological Implications

The refined results not only demonstrate the utility of our system in tracking interaction patterns pivotal for epidemiological studies but also highlight its role in quantifying the exact durations of potential exposure events. This precise data, along with the visual insights from Figure 3 and Figure 4, enables public health officials to deeply understand the dynamics of disease spread within indoor environments, facilitating more effective responses to infectious disease threats [29]. By integrating these findings with real-world epidemiological models, we enhance our capacity to predict and manage outbreaks, tailoring interventions to specific environmental and population dynamics.

The above-mentioned comprehensive analysis underscores the effectiveness of our software in providing actionable insights into indoor human behavior patterns related to disease transmission risks. By integrating real-time tracking data and advanced visualization techniques with epidemiological models, we have developed a robust framework for public health surveillance and intervention strategies. These tools enhance our understanding of disease transmission in indoor environments and support data-driven decisions in public health policy and indoor space management [30].

### 5.3. Future Directions

The research paves the way for further enhancements and explorations. The adaptable nature of the software framework allows for the integration of additional data streams, such as environmental sensors or wearable technologies, to refine the analysis’s granularity and accuracy further. Future initiatives will also explore conducting a comparative analysis of various object detection models to benchmark the performance of YOLOv8 against others, such as Faster R-CNN and SSD, focusing on their efficiency, accuracy, and suitability for real-time tracking in public health scenarios.

A longitudinal study will observe changes in contact rates over extended periods, providing insights into the effectiveness of interventions and behavioral adaptations over time. Additionally, the integration with IoT devices will be examined to enhance data collection by incorporating environmental sensors and smart wearables, thus enriching the context of interaction patterns. We plan to assess the impact of various public health interventions, such as modifications to room layouts and traffic flows, and extend our surveillance system to multiple sites, including schools, offices, and hospitals, to perform multi-site comparisons.

Furthermore, there is significant potential to scale the system to support larger and more complex environments. Such scalability could extend its application to broader contexts such as urban planning, disaster response, and management of large-scale public events, where understanding and controlling crowd dynamics are essential. Employing machine learning algorithms to anticipate disease transmission patterns based on historical data will also be a priority, thus improving the predictive capacity of the system.

Looking forward, the integration of AI-driven predictive analytics and real-time environmental monitoring promises to refine our predictive models and expand their applicability to more complex environments, thus broadening our understanding and control of infectious disease spread. Addressing challenges such as privacy concerns and the need for scalable solutions will be crucial in advancing the field and maximizing the impact of our research on global public health strategies. This proactive approach not only enhances the immediacy and accuracy of our responses but also significantly aids in the management of infectious diseases, ultimately contributing to global health security.

### 5.4. Limitations and Restrictions

There are some limitations or concerns that need to be taken into consideration and addressed in the use of the proposed system’s future developments. First, it should be mentioned that personage detection could be subject to privacy issues even in public spaces, unless proper permission, policies, or regulatory compliance are in place. These regulations may vary from place to place. Considering that the main purpose of the proposed system is to protect public health during major pandemics and infectious disease situations, such surveillance systems can be supported. While faces can be blurred for analytical purposes, the camera setup in indoor spaces should be in accordance with regulations that protect people’s privacy. Second, video capturing for AI-based detection is subject to occluding issues. The results would be more accurate if the individuals did not occlude each other. This challenge could be partially addressed by using multi-camera capturing systems, depth cameras, and AI-based occlusion handling methods. The inclusion of some of these methods are under investigation for the future version of the proposed system. Third, architectural plans, which are used in the transformation processing, should be the same as the built environment from which the videos are captured. In some cases, there are considerable differences between the original design 2D maps and the constructed spaces, which may cause distortion in transforming the motion captures into architectural plans. It is advised that the floor plans are updated for any alteration or intervention. Fourth, real-time processing and transformation to 2D architectural blueprints requires high-processing computation devices. This may limit the wider use of the proposed system in places with camera feeds, as dedicated hardware capacities are needed for real-time processing. Finally, while the proposed system uses low-cost and available camera feeds, the camera setup and data processing will require some fixed and operational costs that need to be taken into consideration. Therefore, a more targeted approach and cost–benefit analysis should be carried out to justify the implementation of the proposed system in public spaces from which capturing contact rate data is very crucial for public health interventions. 

## 6. Conclusions

The technological innovations introduced through this research significantly enhance public health surveillance capabilities. The application of YOLOv8 not only elevates the precision of object detection and tracking but also facilitates real-time operation, which is critical for timely and effective public health interventions. Moreover, the dynamic management of buffer zones and the strategic use of heatmaps and density maps provide a versatile and potent toolset to evaluate and visualize close contacts and interaction densities within various epidemiological scenarios, allowing for adjustments based on real-time observations and evolving health guidelines.

The techniques developed for monitoring people counts and analyzing density within designated polygonal zones offer essential insights into occupancy trends and their association with contact rates. These insights are crucial for both facility management and public health authorities in crafting and enforcing effective social distancing measures and managing indoor occupancy to reduce the transmission risks of infectious diseases.

The insights garnered from this study carry profound implications for public health policies, particularly concerning infectious disease management within indoor settings. The detailed understanding of contact rates, occupancy dynamics, and visual interaction patterns provided by this software tool empowers policymakers and health officials to make informed decisions about the design and execution of infection control strategies. This is especially vital in light of global health emergencies, such as the COVID-19 pandemic, where swift and data-driven decision-making is crucial.

In comparison to previous studies, our research achieves an accuracy of 82% in detecting social distancing using YOLOv8 and advanced visualization techniques, while also providing a framework for analyzing contact rates in indoor environment. Unlike earlier works that focused primarily on detection accuracy, our approach quantifies both social distancing projected in accurate coordinated indoor floorplans and the duration of exposure among individuals. This advancement bridges the gap between detection accuracy and actionable epidemiological insights in built environment, which is essential for informing public health strategies.

In summary, the development of this software tool marks a substantial stride in applying computer vision and spatial analysis to address public health challenges. By seamlessly integrating technological innovations with epidemiological insights and advanced visualizations, this study delivers a vital tool in the repertoire of strategies for managing infectious diseases in indoor environments. As these technologies continue to evolve, they will undoubtedly remain integral to shaping proactive public health responses in the future, enhancing the safety and management of indoor spaces amid ongoing global health threats. This proactive approach not only enhances the immediacy and accuracy of our responses but also significantly aids in the management of infectious diseases, ultimately contributing to global health security.

## Figures and Tables

**Table 1:** Detailed Interaction Data.

Track ID	Interaction with (Track ID)	Overlap Events (Frames)	Overlap Duration (Seconds)
1	10, 3	18, 42	0.60, 1.40
2	4, 12, 13	202, 25, 49	6.73, 0.83, 1.63
3	5, 10, 15	64, 33, 9	2.13, 1.10, 0.30
4	5, 2	3, 125	0.10, 4.17
5	3, 10	1, 2	0.03, 0.07
6	-	-	-
7	8	8	0.27
8	7	17	0.57
9	1, 13	41, 70	1.37, 2.33
10	-	-	-
12	2, 13	15, 33	0.50, 1.10
13	12	17	0.57
15	3	10	0.33

## Data Availability

The data presented in this study are available on request from the corresponding author due to privacy.
